# The Effect of Adding CeO_2_ Nanoparticles to Cu–Ni–Al Alloy for High Temperatures Applications

**DOI:** 10.3390/nano14020143

**Published:** 2024-01-09

**Authors:** Carola Martínez, Camila Arcos, Francisco Briones, Izabel Machado, Mamié Sancy, Marion Bustamante

**Affiliations:** 1 Departamento de Ingeniería de Obras Civiles, Universidad de La Frontera, Temuco 4811230, Chile; marion.bustamante@ufrontera.cl; 2 Departamento de Ingeniería Mecánica y Metalúrgica, Escuela de Ingeniería, Pontificia Universidad Católica de Chile, Santiago 7820436, Chile; 3 Escuela de Mecánica, Pontificia Universidad Católica de Valparaíso, Quilpué 2430120, Chile; francisco.briones@pucv.cl; 4 Departamento de Engenharia Mecatrônica e Sistemas Mecânicos, Escola Politecnica, Universidade de São Paulo, São Paulo 05508-030, Brazil; machadoi@usp.br; 5 Escuela de Construcción Civil, Facultad de Ingeniería, Pontificia Universidad Católica de Chile, Santiago 7820436, Chile; mamiesancy@uc.cl

**Keywords:** Cu–Ni–Al, CeO_2_–NPs, high temperatures, microhardness, impedance, gravimetry measurements

## Abstract

This work presents the effect of CeO_2_ nanoparticles (CeO_2_–NPs) on Cu–50Ni–5Al alloys on morphological, microstructural, degradation, and electrochemical behavior at high temperatures. The samples obtained by mechanical alloying and spark plasma sintering were exposed to a molten eutectic mixture of Li_2_CO_3_–K_2_CO_3_ for 504 h. The degradation of the materials was analyzed using gravimetry measurements and electrochemical impedance spectroscopy. Different characterization techniques, such as X-ray diffraction and scanning electron microscopy, were used to investigate the phase composition, parameter lattice, and microstructure of Cu–Ni–Al alloys reinforced with CeO_2_–NPs. The hardness of the composite was also examined using the Vickers hardness test. Gravimetry measurements revealed that the sample with 1 wt.% CeO_2_–NPs presented the best response to degradation with a less drastic mass variation. Impedance analysis also revealed that by adding 1 wt.% CeO_2_–NPs, the impedance modulus increased, which is related to a lower porosity of the oxide film or a thicker oxide layer. The microhardness also significantly increased, incorporating 1 wt.% CeO_2_–NPs, which reduced with higher CeO_2_–NPs content, which is possibly associated with a more uniform distribution using 1 wt.% CeO_2_–NPs in the Cu–Ni–Al matrix that avoided the aggregation phenomenon.

## 1. Introduction

Fuel cells (FC) are considered a promising technology for being an alternative source of electric power [1]. These devices convert chemical energy into electricity [2,3] and can be classified according to the work temperature [4]. For example, a molten carbonate fuel cell (MCFC), the operational temperature of which is 650 °C, is one of the most efficient FCs, and is contemplated as a carbon capture and storage (CSS) technology because it can capture and convert CO_2_ [5]. The high-temperature fuel cell (600–1000 °C) uses nickel or other non–precious catalytic materials to decrease the electrode cost [6]. The electrode used as the cathode is a porous nickel oxide, where O_2_ and CO_2_ are injected, and on the anode side, nickel aluminum alloy is employed, generally Ni5Al, and supplies H_2_. According to the report by Lee et al. [7], a charge transfer process controls the electrochemical reactions in a slow reaction system and a mass transfer process in a rapid reaction system. Even though the MCFC has been working for decades, there are still problems to resolve due to the high operational temperature. It is essential to consider that the corrosion of the electrodes and equipment that operate at high temperatures can decrease the FC’s service life, as Hacker and Mitsushima proposed [8]. More specifically, few works have focused on the corrosion phenomenon on the anode of MCFC. In this context, Accardo et al. [9] studied the addition of copper [10,11] and cerium oxide nanoparticles (CeO_2_–NPs) to improve the mechanical and electrochemical behavior of the Ni5–Al commercial anode. In this sense, as copper has good electrical and thermal conductivity and mechanical resistance [11,12], the Cu–Ni–Al alloy can be an option for the MCFC anode. On the other hand, some authors reported that incorporating CeO_2_–NPs in the Cu–Ni alloy increased the catalyst performance on the anode for the H_2_ oxidation reaction [13], and incorporating it in the Ni–5Al alloy reduced the creep strain because it can keep the pore structure stable [9]. However, no available data evaluate the micro–macrostructural and corrosion behavior of a Cu–Ni–Al alloy reinforced with CeO_2_ nanoparticles used for the anodes in MCFC. 

Therefore, the effect of nanoparticle addition on the degradation of Cu–50Ni–5Al in the electrolyte Li_2_CO_3_–K_2_CO_3_ (62–38 mol.%) is analyzed in this paper. The microstructural and morphological changes during this process provide valuable insights into the potential use of nanoparticles in the anode.

## 2. Materials and Methods

### 2.1. Sample Obtention

Mechanical alloying was performed using pure powders: Nickel (<10 μm, 99+, Merck, Darmstadt, Germany), Copper (<63 µm, >99%, Sigma-Aldrich, Darmstadt, Germany), and Aluminum (<60 μm, 99.9%, Good Fellow, Hamburg, Germany), which were mechanically alloyed (MA) in a Planetary mill PQ4 Across International, obtaining powder compositions of Cu–50Ni–5Al (wt.%). The milling conditions included a ball–a–powder ratio (BPR) of 10:1 and 2 wt.% stearic acid as a control agent under an inert Ar atmosphere. The milling time used was 100 h effective and there was an on/off cycle of 30/15 min at a speed of 350 r.p.m. Subsequently, 1, 3, and 5 wt.% of the nanoparticles CeO_2_ (CeO_2_–NPs) (<25 nm, >99.9%, Sigma-Aldrich, Hamburg, Germany) were added to the alloy using Mixer Y–type Astecma for 1 h. Table 1 shows the chemical composition of Cu–50Ni–5Al + xCeO_2_ (wt.%) alloys.

**Figure 1:**
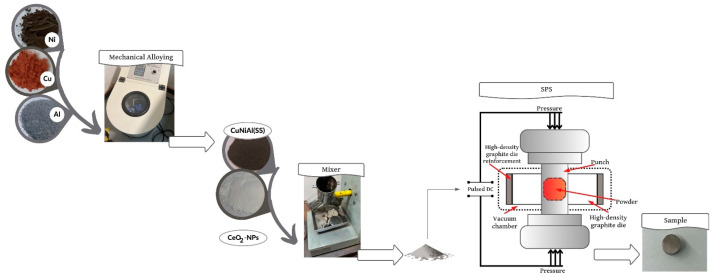
Sample preparation (powder mixture) and schematic representation of the SPS system (consolidation).

Cu–50Ni–5Al without and with the CeO_2_–NPs samples were consolidated by Spark Plasma Sintering (SPS) using a Fuji Electronic Industrial Co model DR. SINTER^®^ SPS1050. The disks were 10 mm in diameter and 7 mm in thickness, and were produced using a high–density graphite die. The samples were heated from room temperature to 800 °C at a heating rate of 100 °C min^−1^, applying a pressure of 50 MPa simultaneously during the heating and holding time of 5 min at the sintering temperature. The entire SPS process was kept under a vacuum of approximately 20 Pa. Finally, the samples were free–cooled to room temperature at a cooling rate of roughly 10 °C s^−1^ in the SPS chamber. Figure 1 shows the sample preparation (powders) diagram by mechanical alloying and mechanical mixing and a schematic representation of the consolidation process by the SPS system.

The metal samples were polished using sandpaper from #800 to #4000 and then with colloidal silica suspension to reveal their microstructure. The polished samples were rinsed with ethanol for 10 min in a bath cleaning sonicator. Afterward, the samples were cleaned with distilled water and dried at room temperature. 

### 2.2. Gravimetric Measurements

The Cu–50Ni–5Al samples were immersed in molten eutectic Li_2_CO_3_–K_2_CO_3_ (62–38 mol.%) [14,15] for 504 h (21 days) in an aerated atmosphere or not–controlled medium at 550 ± 5 °C. The gravimetric measurements of samples were carried out as described previously by Arcos et al. [16]. The samples were removed and cleaned to eliminate the deposits and corrosion products on the surface. The bulk samples were submerged in hot distilled water (~100 °C) in a sonicator bath (Elma D–78224 Singen/Htw) for 30 min. Subsequently, the samples were dried with hot air and weighed until they reached a constant value, as reported by the ASTM G1–03 [17]. The average mass (%) was calculated using Equation (1).

(1) $$\frac{m_{i}-m_{f}}{m_{i}}\times 100$$

where $m_{i}$ and $m_{f}$ are the initial and final sample masses at different exposure times.

### 2.3. Morphological and Chemical Characterization

The porosity was determined through Archimedes’ method, according to the Standard Test ASTM C373–88 [18]. To reveal the microstructure, an etching was employed: 5 g of Fe_3_Cl, 10 mL of HCl, and 100 mL of distilled water for 8 s. A field–emission scanning electron microscope (FE-SEM), QUANTA FEG 250, was used to obtain the samples’ images. 

X-ray diffraction (XRD) was implemented using Bruker D2 PHASER with Cu–*K*α radiation to characterize the material’s structure. The diffraction patterns were recorded from 2θ between 40° and 100° with a 0.02° step and counting time of 1 s/step.

### 2.4. Mechanical Characterization

The microhardness of the samples was calculated by a micro–Vickers durometer, Wilson^®^ VH1150 Macro Vickers Hardness Tester, under 0.3 kgf of force before the gravimetric measurements.

### 2.5. Electrochemical Measurements

The electrochemical behavior of the Cu–50Ni–5Al samples was studied using open circuit potential and electrochemical impedance spectroscopy (EIS) measurements in the molten Li_2_CO_3_–K_2_CO_3_ (62:38 mol.%) as an electrolyte at 550 ± 5 °C under an aerated atmosphere. The electric contact for the working electrode, the Cu–50Ni–5Al samples, was performed using conductive silver printing ink (resistivity 5–6 µΩ cm) around the sample and copper wire of 25 cm in length. In addition, a Pt wire that was 25 cm in length was used as a counter electrode, and an Ag wire that was 25 cm in length and placed inside a quartz glass tube with a porous plug in the tip was used as the reference electrode. The electrochemical measurements were carried out with a Potentiostat Solartron Analytical. 

## 3. Results and Discussion

### 3.1. Gravimetric Measurements

**Figure 2:**
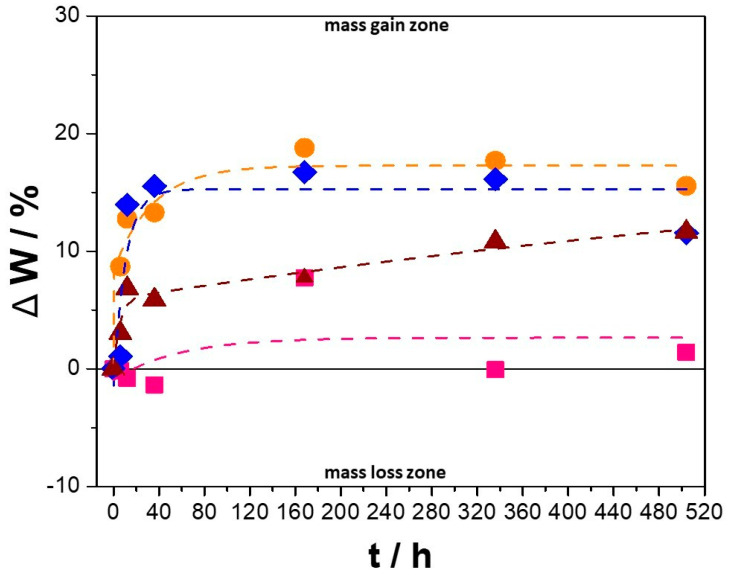
The variation of weight of Cu–50Ni–5Al samples after 504 h of exposure to molten carbonates. (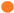) 0 wt.% CeO_2_–NPs, (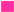) 1 wt.% CeO_2_–NPs, (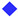) 3 wt.% CeO_2_–NPs, (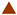) 5 wt.% CeO_2_–NPs.

Figure 2 shows the effect of CeO_2_–NPs on the weight gain of the Cu–50Ni–5Al samples after exposure to molten carbonates, revealing a reduction in the weight by adding CeO_2_–NPs. In addition, Figure 2 shows that during the initial stage, the weight increased rapidly for all the Cu–50Ni–5Al samples, which was lower for Cu–50Ni–5Al + 1 wt.% CeO_2_–NPs, reaching a maximum weight gain of only 1.2% at 504 h of exposure. For a longer exposure time, the weight gain reaches a stationary state associated with a passive oxide layer [16] formed after 80 h for Cu–50Ni–5Al + 3 wt.% CeO_2_–NPs, and after 160 h for Cu–50Ni–5Al + 0 wt.% CeO_2_–NPs. It should be noted that with 5 wt.% CeO_2_–NPs, the weight gain did not reach a plateau of up to 504 h of exposure, like the sample with 3 wt.% CeO_2_–NPs. Therefore, the sample that suffered the least degradation at high temperatures was 1 wt.% CeO_2_–NPs, which could be attributed to the homogeneous distribution of the CeO_2_–NPs.

### 3.2. Microstructural and Chemical Characterization

**Figure 3:**
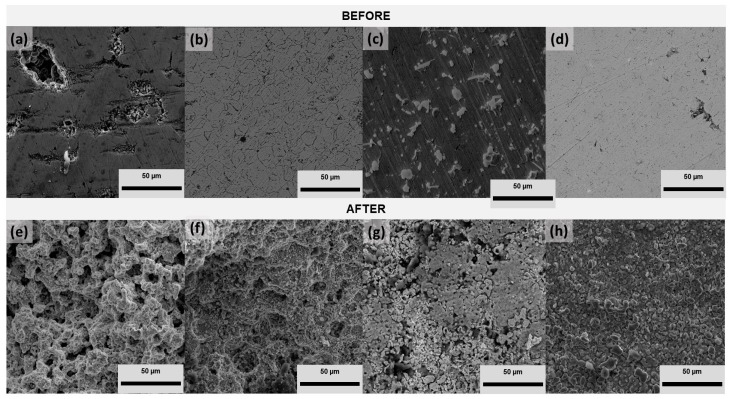
FE–SEM images before Cu–50Ni–5Al: (**a**) 0 wt.% CeO_2_–NPs, (**b**) 1 wt.% CeO_2_–NPs, (**c**) 3 wt.% CeO_2_–NPs, (**d**) 5 wt.% CeO_2_–NPs, and after gravimetric measurements Cu–50Ni–5Al, (**e**) 0 wt.% CeO_2_–NPs, (**f**) 1 wt.% CeO_2_–NPs, (**g**) 3 wt.% CeO_2_–NPs, and (**h**) 5 wt.% CeO_2_–NPs.

Figure 3 shows the morphology of the samples before and after 504 h of exposure to molten carbonate (Li_2_CO_3_–K_2_CO_3_ 62–38 mol.%) in the aerated atmosphere. Before exposure, porosity can be observed for all the samples, agreeing with the analysis performed. For example, Cu–50Ni–5Al had 16 ± 0.8% porosity, which reduced the addition of CeO_2_–NPs. The Cu–50Ni–5Al + 1 wt.% CeO_2_–NPs samples had 1.0 ± 0.3% porosity, Cu–50Ni–5Al + 3 wt.% CeO_2_–NPs had 2.0 ± 0.2% porosity, presenting some spots more lightly over the surface, and Cu–50Ni–5Al + 5 wt.% CeO_2_–NPs had 1.0 ± 0.1% porosity. One reason for this phenomenon is that nanoparticles can easily remain in the pores and voids of the nanocomposite matrix due to their small size [19]. After exposure, a strong surface modification was observed for all the samples, possibly due to the corrosion product formation, which could be a passive film, as suggested by the gravimetric measurements. Ren et al. [20] studied a Cu–35Ni–10Al alloy in molten carbonate (Li_2_CO_3_–K_2_CO_3_ 62–38 mol.%) for 48 h in an aerated atmosphere, reporting the formation of porous corrosion products composed mainly of Al_2_O_3_ and Ni–Al oxides, which is in agreement with the SEM images of Cu–50Ni–5Al (see Figure 3a), which present a porous surface after exposure. 

**Figure 4:**
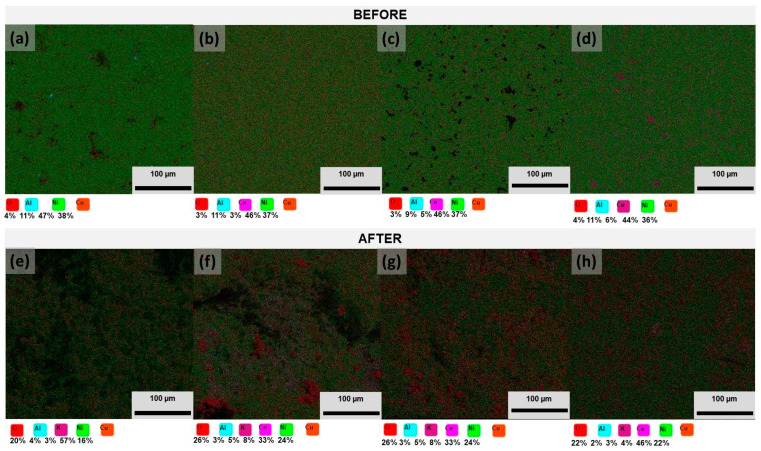
EDS surface mapping before Cu–50Ni–5Al: (**a**) 0 wt.% CeO_2_–NPs, (**b**) 1 wt.% CeO_2_–NPs, (**c**) 3 wt.% CeO_2_–NPs, (**d**) 5 wt.% CeO_2_–NPs and after gravimetric measurements Cu–50Ni–5Al; (**e**) 0 wt.% CeO_2_–NPs, (**f**) 1 wt.% CeO_2_–NPs, (**g**) 3 wt.% CeO_2_–NPs, and (**h**) 5 wt.% CeO_2_–NPs.

Figure 4 presents the EDS results before and after exposure to analyze the chemical composition of the sample’s surface. Before exposure, the alloy’s surface is very similar for all the samples, revealing a homogeneous distribution of all the elements (Cu, Ni, Al, and O). However, Cu–50Ni–5Al has some Al spots, and Cu–50Ni–5Al + 3 wt.% CeO_2_–NPs have some zones not identified by the mapping, which could be Li because it has deficient energy and is difficult to detect. Cu–50Ni–5Al+ 5 wt.% CeO_2_–NPs present some nanoparticles agglomeration (CeO_2_) corresponding to the element Ce. Frattini et al. [21] observed the same effect when adding small amounts of ZrO_2_–NPs in the Ni–Al alloy, although, with the increase in the amount to 10% ZrO_2_–NPs, the distribution of the NPs becomes homogeneous. After exposure, the quantity of oxygen (O) increased significantly for all the samples, which can be attributed to the oxide formation on the surface, as proposed above. Nevertheless, potassium (K) was also found on the surface, a component of the molten carbonates (Li_2_CO_3_–K_2_CO_3_), revealing the possible formation of a deposit. According to Gonzalez–Rodriguez et al. [22], the Ni–50Al alloy was immersed in Li_2_CO_3_–K_2_CO_3_ (62:38 mol%) for 100 h in static air at 650 °C. They reported that the main corrosion products were Ni, Al, and K, such as NiO, Al_2_O_3_, LiAlO_2_, and LiKCO_2_. Also, Ren et al. [20] described mainly Al_2_O_3_ and Cu_2_O as the corrosion products of Cu–35Ni–10Al exposure to Li_2_CO_3_–K_2_CO_3_ (62:38 mol%) during 1 h in air at 650 °C. Accardo et al. [9] also proposed that CeO_2_–NPs could migrate from the alloy to the electrolyte. EDS analysis revealed a slight decrease in the Ce content for the Cu–50Ni–5Al + 5 wt.% CeO_2_–NPs sample. For the other samples, the Ce content increases, which can be associated with diffusion from the bulk to the surface of the molten carbonates.

**Figure 5:**
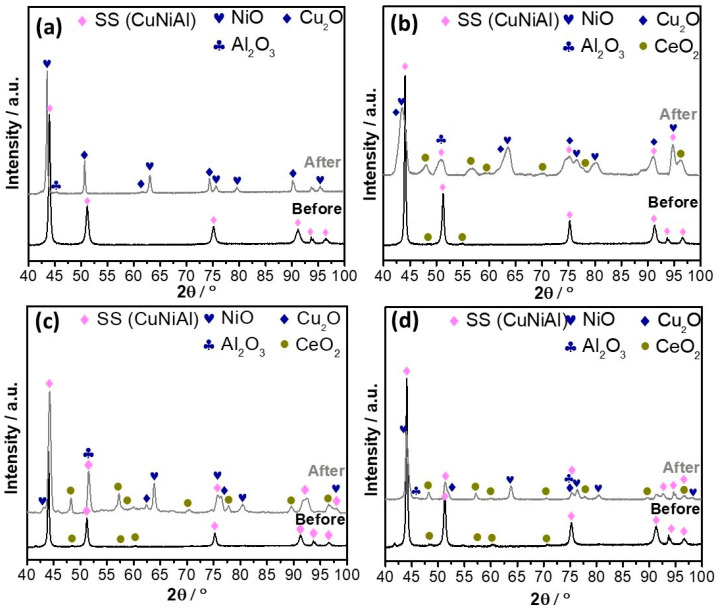
XRD patterns of Cu–50Ni–5Al samples: (**a**) 0 wt.% CeO_2_–NPs, (**b**) 1 wt.% CeO_2_–NPs, (**c**) 3 wt.% CeO_2_–NPs, (**d**) 5 wt.% CeO_2_–NPs before and after gravimetric measurements.

Figure 5 compares the XRD patterns recorded before and after exposure to Li_2_CO_3_–K_2_CO_3_ (62:38 mol%). Before exposure, the reflections were identified as corresponding to typical fcc structures (Fm-3m). No peaks were associated with Ni or Al, indicating that the solid solution Cu–Ni–Al obtained by mechanical alloying is maintained post–sintering by SPS. The samples reinforced with CeO_2_–NPs show low-intensity reflections associated with CeO_2_ (Fm-3m; JCPDS 010750076). The lattice parameter of the Cu-Ni–Al alloys without CeO_2_–NPs is 0.358 nm, which remains constant when incorporating the different CeO_2_–NPs. This indicates that the CeO_2_–NPs do not react with the Cu–Ni–Al matrix in the consolidation process because the SPS technique is a fast method for sintering [23]. After 21 days of exposure to the Li_2_CO_3_–K_2_CO_3_, peaks associated with NiO (Fm-3m; JCPDS 010731519), Cu_2_O (Pn-3m; JCPDS 010751531), and Al_2_O_3_ (R-3c; JCPDS 010772135) can be seen in all the samples. In addition, the intensities of the reflections associated with CeO_2_–NPs can be seen, which can be attributed to the fact that the CeO_2_–NPs migrate to the surface, as reported by Accardo et al. [9]. Note that the increase in the intensity of the reflections associated with CeO_2_ is much lower for the sample with 5 wt.% of CeO_2_–NPs than in the samples with 1 wt.% and 3 wt.% of CeO_2_–NPs, possibly because the Ce content decreased on the sample surface, confirming what was previously mentioned in Figure 4.

### 3.3. Mechanical Properties

**Figure 6:**
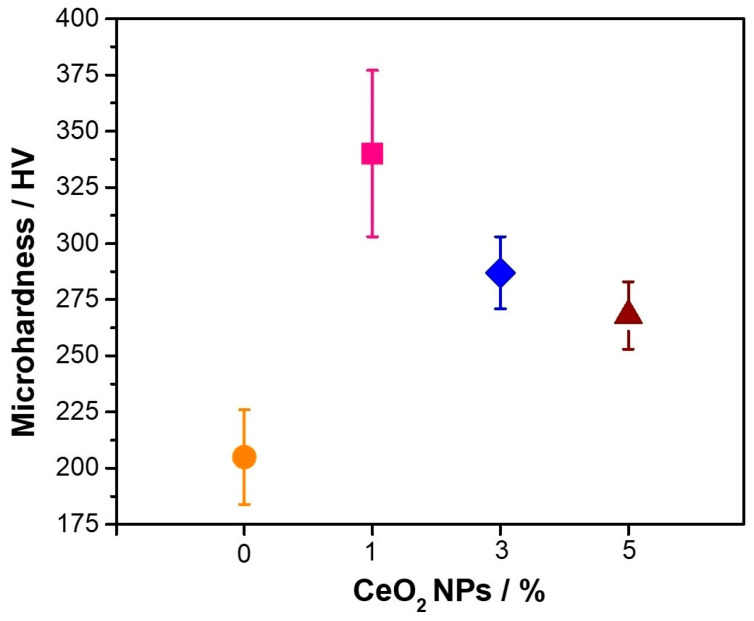
Microhardness of the samples Cu–50Ni–5Al: (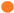) 0% wt.% CeO_2_–NPs, (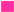) 1% wt.% CeO_2_–NPs, (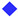) 3% wt.% CeO_2_–NPs, and (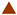) 5% wt.% CeO_2_–NPs before exposure.

Figure 6 shows the hardness of the Cu–50Ni–5Al + x wt.% CeO_2_–NPs samples before exposure. The results indicate that the sample with 0% CeO_2_–NPs presents the lowest hardness value, corresponding to 205 ± 21 HV_0.3_. This can be attributed to the higher porosity of the sample, which reaches 16%, by incorporating different amounts of CeO_2_–NPs in the sample. Cu–50Ni–5Al +1 wt.% CeO_2_–NPs perform better due to the CeO_2_–NPs allowing a decrease in the porosity and a homogenous distribution of CeO_2_–NPs in the matrix, as mentioned above. However, if the concentration of CeO_2_–NPs exceeds 1 wt.%, they agglomerate at grain boundaries, reducing the hardness value, as seen in Figure 4. Zawrah et al. [24] concluded that adding Al_2_O_3_–NPs to pure Cu improves hardness due to their uniform distribution. The improved hardness can be attributed to the relative contribution of the Orowan strengthening effect, mainly when the reinforcement size is less than 100 nm [25]. The CeO_2_–NPs are very small and hard, impeding the movement of dislocations in the Cu–50Ni–5Al matrix, leading to an improvement in the hardness of the microstructure.

### 3.4. Electrochemical Measurements

**Figure 7:**
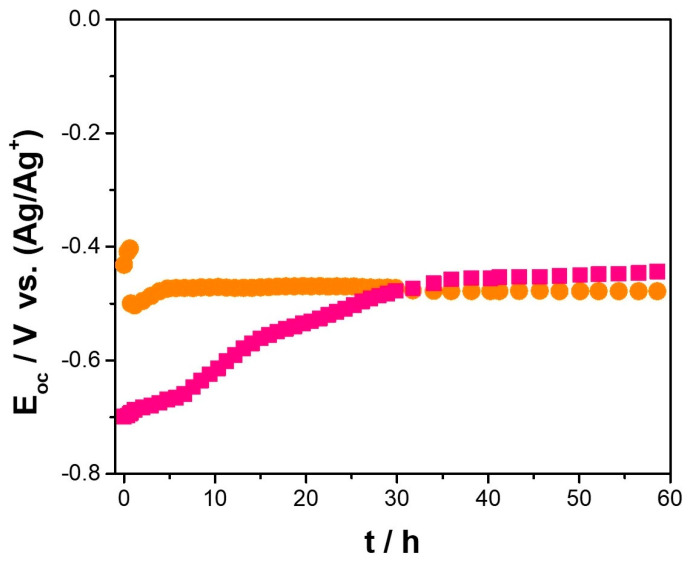
Open circuit potential variation of (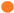) Cu–50Ni–5Al and (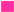) Cu–50Ni–5Al + 1 wt.% CeO_2_–NPs exposure to Li_2_CO_3_–K_2_CO_3_ at 550 °C and aerated atmosphere over time.

Figure 7 shows the effect of the addition of 1 wt.% CeO_2_–NPs to the open circuit potential (E_OC_) of Cu–50Ni–5Al after exposure to Li_2_CO_3_–K_2_CO_3_ at 550 °C and an aerated atmosphere. After a shorter exposure time, the E_OC_ was shifted to more negative values by incorporating CeO_2_–NPs, suggesting an activation of the corrosion phenomena. However, for a longer exposure time, the E_OC_ reached similar values to the sample without CeO_2_–NPs, which can be associated with a stable oxide layer formed on the metal surface. Meléndez-Ceballos et al. [26] studied a Ni porous sample coated by CeO_2_–NPs using the atomic layer deposition in Li_2_CO_3_–K_2_CO_3_ at 650 °C in a CO_2_/air 30/70 vol.% atmosphere. The authors determined an initial potential close to -0.76 V vs. Ag/Ag^+^; the reference electrode is a silver wire submerged in Ag_2_SO_4_ (10^−1^ mol kg^−1^) saturated in Li_2_CO_3_–K_2_CO_3_, which was shifted to more positive values as a function of exposure time, attributed to a delay in the Ni oxidation process due to the presence of CeO_2_–NPs film. 

**Figure 8:**
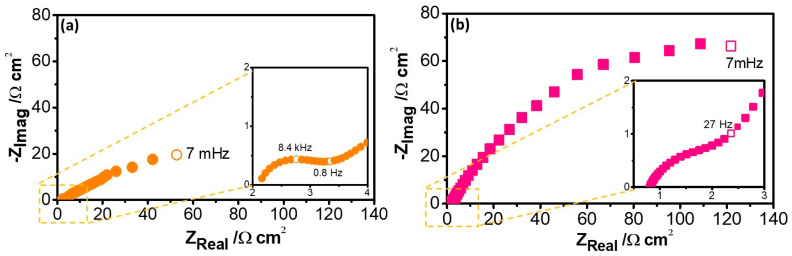
Nyquist diagrams of (**a**) Cu–50Ni–5Al and (**b**) Cu–50Ni–5Al + 1 wt.% CeO_2_–NPs exposure 1 h to Li_2_CO_3_–K_2_CO_3_ at 550 °C, aerated atmosphere, and E = E_OC_.

Figure 8 shows the Nyquist diagrams of Cu–50Ni–5Al after 1 h of exposure to Li_2_CO_3_–K_2_CO_3_ in an aerated atmosphere at E = E_OC_ and 550 °C, revealing a significant increase in the impedance modulus due to the incorporation of 1 wt.% CeO_2_–NPs, which could be related to the formation of a passive oxide layer on the alloy, as previously mentioned. As can be seen at E = E_OC_, the impedance responses reveal two time constants at high and low frequency ranges (HF and LF), which can be associated with the cathodic current, not only involving the capacitance of the electric double layer (C_dl_) and the oxygen reduction reaction, but also the formation of an oxide layer due to the alloy dissolution. Different equivalent circuits have been proposed to represent the physical model, which can be composed of capacitors, resistances, and constant phase elements (CPE) related to the heterogeneity of the surface [27].

**Figure 9:**
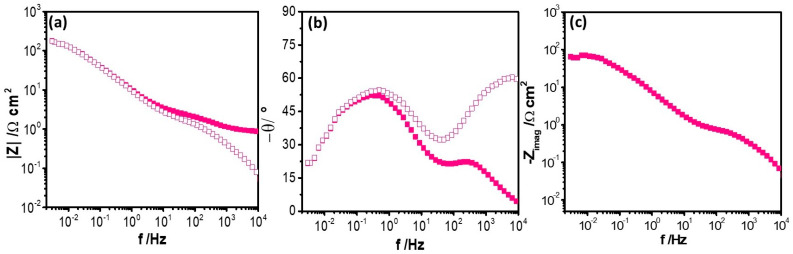
(**a**,**b**) Effect of correction of electrolyte resistance on Bode plots and (**c**) variation of the imaginary part of the impedance of Cu–50Ni–5Al + 1% wt.% CeO_2_–NPs exposure 1 h to Li_2_CO_3_–K_2_CO_3_ in aerated atmosphere at 550 °C and E = E_OC_.

Figure 9 shows the Bode plots of Cu–50Ni–5Al + 1 wt.% CeO_2_–NPs after 1 h of exposure to Li_2_CO_3_–K_2_CO_3_ in an aerated atmosphere at E = E_OC_ and 550 °C, as a representative example of the study system. Figure 9 reveals a capacitive response with two or three time constants at all frequency ranges, possibly associated with the formation of an oxide film and oxygen reduction reaction. Additionally, the Bode plots revealed a higher impedance modulus at the LF range when CeO_2_–NPs were added, which can be related to the polarization resistance of the system, suggesting an enhancement of the corrosion resistance due to the incorporation of the CeO_2_–NPs to the Cu–50Ni–5Al matrix [27,28,29]. Moreover, Figure 9 shows the non–corrected (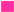) and corrected Bode plots by electrolyte resistance (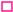), revealing that this effect is mainly in the high frequency range.

Figure 9c shows the variation of the imaginary part of the impedance of Cu–50Ni–5Al + 1 wt.% CeO_2_–NPs as a function of frequency after 1 h of exposure to Li_2_CO_3_–K_2_CO_3_ in an aerated atmosphere at E = E_OC_ and 550 °C, revealing a constant phase element (CPE) behavior in the MF range, with a negative slope (α) that varied between −0.33 ± 0.01 for Cu–50Ni–5Al and -0.65 ± 0.004 for Cu–50Ni–5Al + 1% wt.% CeO_2_–NPs, which can be related to the oxide film formed on the metal surface and described by the following relation, as reported by Orazem and Tribollet [30], Tribollet et al. [31], and Hirschorm et al. [32].

(2) $$Z_{oxide}=\int_{0}^{\delta }\frac{\rho \left(\gamma \right)}{1+j\omega \rho \left(\gamma \right)\varepsilon \left(\gamma \right)\varepsilon_{0}}d\gamma$$

The authors proposed the power-law model (PLM) to analyze the film properties using Equation (3):

(3) $$Z\left(\omega \right)=g\frac{\delta \rho_{\delta }^{1-\alpha }}{{\left(\rho_{0}^{-1}+j\omega \varepsilon \varepsilon_{0}\right)}^{\alpha }}$$

In this case, *α* is the slope in the Log *Z_Imag_* vs. Log *f* plots, *ε* represents the dielectric constant of the oxide layer formed on the metal alloy, *ε*_0_ is the vacuum permittivity that is equal to 8.85 × 10^−14^ F·cm^−1^, and g is a numerical coefficient close to 1 when *α* is 1, which can be estimated using the following equation:

(4) $$g=1+2.88\;{\left(1-a\right)}^{2.375}$$

In addition, *ρ*_0_ and *ρ*_δ_ represent the lower and upper limits in the frequency range where CPE behavior is observed. The *Q* value corresponds to a CPE parameter that can be determined using the following equation:

(5) $$Q=\frac{{\left(\varepsilon \varepsilon_{0}\right)}^{\alpha }}{g\delta \rho_{\delta }^{1-\alpha }}$$

The graphical method of the impedance data allowed us to estimate the CPE parameters for the Cu–50Ni–5Al Q coefficient of 1.62 × 10^−3^ F·cm^−2^·s^–(1−α)^ and |α| value of 0.60, and for the Cu–50Ni–5Al + 1% wt.% CeO_2_–NPs, the Q coefficient of 6.95 × 10^−3^ F·cm^−2^·s^–(1−α)^ and |α| value of 0.67. Furthermore, electrolyte resistance (*R*e) was determined by the graphical method, finding values close to 2.7 Ω cm^2^ for Cu–50Ni–5Al and 0.84 Ω cm^2^ for the Cu–50Ni–5Al + 1% wt.% CeO_2_–NPs samples. Those parameters reveal that the addition of CeO_2_–NPs to Cu–50Ni–5Al improves the corrosion resistance, possibly due to a lower porosity of the oxide film or a thicker oxide layer.

## 4. Conclusions

The Cu–50Ni–5Al alloys were obtained by mechanical alloying and the SPS process, which allowed the addition of CeO_2_–NPs into the metal matrix. The samples were exposed to molten carbonate (Li_2_CO_3_–K_2_CO_3_), consequently forming corrosion products over the surface, which were analyzed by SEM-EDS and corroborated by X-ray diffraction. The addition of CeO_2_–NPs in the Cu–50Ni–5Al matrix reduced the mass variation over time, mainly using 1% wt. CeO_2_, reaching a maximum weight gain of only 1.2% at 504 h of exposure. The corrosion products were composed of nickel oxide, aluminum oxide, and copper oxide in all the alloys, regardless of the amount of NPs incorporated.

Moreover, the microhardness significantly increased for the alloy containing 1 wt.% of CeO_2_–NPs, reaching a hardness value of 340 HV_0.3_. Furthermore, the impedance analysis revealed that the samples with 1 wt.% of CeO_2_–NPs in the molten carbonates in an aerated atmosphere had a higher impedance modulus, possibly due to the lower porosity of the oxide film or a thicker oxide layer. 

Therefore, the alloys that showed better mechanical behavior and higher corrosion resistance were Cu–50Ni–5Al + 1 wt.% CeO_2_–NPs, which have a promissory use at high temperatures.

## Figures and Tables

**Table 1:** Chemical composition of the Cu–50Ni–5Al + xCeO_2_ (wt.%) alloys.

Sample	Cu	Ni	Al	CeO_2_
0 wt.% CeO_2_–NPs	Bal.	50	5	0
1 wt.% CeO_2_–NPs	Bal.	50	5	1
3 wt.% CeO_2_–NPs	Bal.	50	5	3
5 wt.% CeO_2_–NPs	Bal.	50	5	5

## Data Availability

The data presented in this study are available on request from the corresponding author.
